# Mercury Toxicity and Contamination of Households from the Use of Skin Creams Adulterated with Mercurous Chloride (Calomel)

**DOI:** 10.3390/ijerph120910943

**Published:** 2015-09-02

**Authors:** Lori Copan, Jeff Fowles, Tracy Barreau, Nancy McGee

**Affiliations:** 1 California Department of Public Health, Environmental Health Investigations Branch, Richmond, CA 94706, USA; E-Mails: Jeff.Fowles@cdph.ca.gov (J.F.); Tracy.Barreau@cdph.ca.gov (T.B.); 2 California Department of Toxic Substances Control, Enforcement and Emergency Response Division, Sacramento, CA 95826-3200, USA; E-Mail: Nancy.McGee@dtsc.ca.gov

**Keywords:** calomel, inorganic mercury poisoning, mercurous chloride, mercury health-based guidance values, mercury toxicity children, residential mercury contamination, skin lightening cream

## Abstract

Inorganic mercury, in the form of mercurous chloride, or calomel, is intentionally added to some cosmetic products sold through informal channels in Mexico and the US for skin lightening and acne treatment. These products have led to multiple cases of mercury poisoning but few investigations have addressed the contamination of cream users’ homes. We report on several cases of mercury poisoning among three Mexican-American families in California from use of mercury-containing skin creams. Each case resulted in widespread household contamination and secondary contamination of family members. Urine mercury levels in cream users ranged from 37 to 482 µg/g creatinine and in non-users from non-detectable to 107 µg/g creatinine. Air concentrations of up to 8 µg/m^3^ of mercury within homes exceeded the USEPA/ATSDR health-based guidance and action level of <1.0 μg/m^3^. Mercury contamination of cream users’ homes presented a multi-pathway exposure environment to residents. Homes required extensive decontamination, including disposal of most household items, to achieve acceptable air levels. The acceptable air levels used were not designed to consider multi-pathway exposure scenarios. These findings support that the calomel is able to change valence form to elemental mercury and volatilize once exposed to the skin or surfaces in the indoor environment.

## 1. Introduction

Inorganic mercury salts, such as mercurous chloride, mercuric chloride, and mercuric oxide, have been found in skin-lightening and anti-acne cosmetic products imported into the US internationally from small scale, unregulated suppliers and through the Internet. The use of these mercury-adulterated skin and face creams for the purposes of skin lightening has been described and reviewed, with case reports from Africa, Europe, Mexico, Asia, and the US [1]. Various researchers and health departments across the US have documented the commercial availability of mercury-containing skin-lightening preparations in their state [2,3,4,5,6,7,8,9]. Hamann and colleagues [3] reported that 6% of 549 creams purchased online or in stores in the US or various parts of Asia contained mercury in excess of 1000 ppm. In their survey, a crude detector with a minimum detection limit of 200 ppm mercury was used to screen products.

Clinical case studies in Mexican women have found mercurous chloride (HgCl) added to facial creams at concentrations of up to 5.9% by weight (59,000 ppm) resulted in elevated urinary mercury of up to 1876 µg/g creatinine [10]. Despite the wide-spread documentation of mercury poisoning in users of skin-lightening creams, and the vast array of literature on the availability of such preparations [3], little is known about the dynamics of inorganic mercury salts within the residential setting, and how these may affect long-term exposure. By comparison, residential contamination and cleanup of elemental mercury is complex, but well-documented and better understood. A study conducted by Spedding and Hamilton [11] using a model home found wide variations in elemental mercury deposition and desorption rates on surfaces commonly encountered in a house with polyvinyl chloride flooring and water-based paint surfaces having the highest affinities for mercury. Decontamination standards for elemental mercury, first established in 2000 [12], are based on health endpoints from exposure to mercury vapor. These action levels may not be suitable in cases of household contamination from inorganic mercury salts where exposure to mercury is through both elevated mercury vapor levels and dermal absorption from contaminated surfaces. 

We report on several cases (Table 1) in California where mercury-adulterated cosmetics resulted in demonstrable exposures to mercury with clinical signs of toxicity not only to the cream user, but also to other family members, through a surprisingly pervasive mercury contamination of the cream users’ households.

## 2. Methods 

The studies described in this manuscript were the result of public health emergency investigations into exposures and poisonings of multiple household members from inorganic mercury. Due to its nature, human IRB approval was not necessary. All patient records were anonymized and de-identified prior to statistical analysis. Descriptive statistical analyses were performed using Microsoft Excel.

Urine mercury concentrations were measured using the method for mercury by inductively coupled plasma mass spectrometry (SW-846 EPA Method 6020A).

Measurements of indoor air and personal belongings were collected predominantly using a Lumex^®^ RA-915 + or a Lumex^®^ RA-915 Light mercury vapor analyzer (Ohio Lumex) [13]. The Lumex^®^ RA-915 + and RA-915 Light’s range of detection for mercury in ambient air is 0.002 µg/m^3^–100 µg/m^3^ and 0.10 µg/m^3^–200 µg/m^3^, respectively (Ohio Lumex). In addition to a Lumex, measurements of indoor air mercury levels at the 2014 family’s home were measured with a Jerome J405 Mercury Vapor Analyzer. The range of detection of the Jerome is 0.5 µg/m^3^ to 999 µg/m^3^.

Breathing zone and floor level air measurements were collected throughout each home. The inside of the washing machine and dryer were also measured. Personal items (e.g., furniture, clothing, beauty products, cleaning rags, towels, vacuum cleaner) were placed in plastic bags in the sun to warm (approximately 10 min), allowing vapors to build-up in the headspace, prior to recording measurements. 

## 3. Results 

### 3.1. Summary of Cases

In 2014, following two hospitalizations, a 20-month-old was diagnosed with mercury poisoning. The baby exhibited hypertension, refusal to walk, irritability, difficulty sleeping, and required a nasogastric feeding tube for poor appetite. The baby’s mother used an artisanal skin-lightening cream from Mexico. The baby’s mercury urine level was 52 µg/g creatinine and the most likely exposure route was through physical contact with the mother or from contact with contaminated household items. Many of the family’s personal belongings were discarded because of mercury contamination. The cream used by the mother was subsequently tested by the California Department of Public Health (CDPH) Food and Drug Laboratory and found to contain 38,000 ppm of mercury. As a result of media outreach and contact tracing of other people known to use the cream, five other contaminated households were identified. Eleven out of 24 people tested in these households had elevated urine mercury levels; of those, five were cream users and six were not cream-users. 

In 2013, following several emergency room visits, consultations with a neurologist, and a week-long hospitalization, a 17-year-old was admitted to a pediatric intensive care unit and remained hospitalized for almost a month after using a non-commercial, artisanal cream from Mexico for acne. His symptoms progressed rapidly from weakness in his legs to involuntary muscle twitching. Later he developed severe back pain, diffuse and visible muscle twitching of the extremities, tongue, and lips, unsteady gait, delirium, agitation, sleep disturbances, profuse sweating, persistent tachycardia, and hypertension. After two weeks in the hospital, the adolescent’s mercury urine level was tested and found to be 144 µg/g creatinine from a spot urine and 208 µg/g creatinine from a 24-h urine. At this point, a diagnosis of mercury poisoning was determined and chelation therapy initiated. He had only been using the acne cream twice a day for about six weeks before the onset of symptoms. Eleven family members were found to have elevated mercury levels and almost all furniture and personal belongings in the home were discarded as hazardous waste. The creams were later tested by CDPH and found to contain between 96,000 ppm to 210,000 ppm of mercury. 

In 2010, a 39-year-old woman and her four-year-old child were found to have elevated urine mercury levels after participating in a health study. The woman had 482 µg/g creatinine of mercury in urine and the four-year-old child had 107 µg/g creatinine. A clinical examination showed that the woman experienced mild to moderate symptoms of mercury toxicity, including numbness and tingling in her hands and lips, dizziness, forgetfulness, headaches, depression, irritability, and anxiety. The four-year-old appeared to be developing normally with no clinical symptoms of mercury toxicity. The woman had used an artisanal skin-lightening cream from Mexico for three years to fade freckles and age spots but her child did not use the cream. An additional 21 friends and family were assessed for mercury poisoning and five homes were inspected for contamination. The creams used were found to contain between 20,000 ppm and 57,000 ppm of mercury. 

**Table 1:** Cases in three family units, with individual urinary mercury levels and mercury cream concentrations.

	Cream User	Ages	Sex	Hg Urine (µg/g cr.)	Frequency of Use	Cream Hg Level (ppm)
**Family1, 2014**	N (index)	20 mos	F	52	none	
	Y	35	F	21	unknown	28,000–38,000 ^a^
	N	32	M	2.5	none	
	N	64	F	6	none	
**Family 2, 2013**	Y (index)	17	M	208	Twice daily × 6wks	96,000–210,000 ^a^
	N	4	M	31	none	
	N	?	F	16	-	
	N	2	F	16	none	
	N	11	M	14	none	
	Y	33	M	14	Infrequent	96,000–210,000
	N	10	F	13	none	
	N	9	M	12	none	
	N	6 mos	F	10	-	
	N	34	F	10	none	
	N	28	M	6	none	
**Family 3, 2010**	Y (index)	39	F	482	Twice daily × 4 yrs	56,000 ^b^
	Y	39	M	132	At bedtime	56,000
	N	4	F	107	none	
	N	8	M	21	none	
	N	14	F	ND **	none	

Note: cr. = creatinine; ^a^ Method described by Gordon A Vrdoljak, Peter T Palmer, Richard Jacobs, Bahman Moezzi, Abstracts, 248th National Meeting of the American Chemical Society, San Francisco, CA, USA, 10–14 August 2014; American Chemical Society: Washington, DC, USA, 2014; ACS ANYL 302; ^b^ Mercury concentrations were measured using the method for mercury by inductively coupled plasma mass spectrometry (SW-846 EPA Method 6020A); ** ND = not detected; limit of quantification = 0.5 µg/g cr.

Table 1 shows the surprising prevalence and magnitude of elevated urine mercury concentrations in family members who were not users of the creams. The 95th percentile of urine mercury levels in Mexican-Americans (2011–2012) from the Centers for Disease Control and Prevention [14], Fourth National Report on Human Exposure to Environmental Chemicals is 1.75 µg/g creatinine. Urinary mercury concentrations were also above CDC level of concern (2014) of 10 µg/g creatinine in 11 of the 15 non cream users, with the highest at the alarming level of 107 µg/g creatinine. All of the cream users, regardless of frequency of use, had urinary mercury well above this level of concern. Table 2 describes the geometric mean and ranges of urine mercury levels in frequent, infrequent, and non-users of the creams.

**Table 2:** Summary of mercury concentration in creams and urine mercury levels in three family units.

Family (Concentration of Hg in Cream in)	Geometric Mean (GSD) or Single Values of Hg in Urine (µg/g cr.)	Range (µg/g cr.)
**Family 1 (2014)** (28,000–38,000 ppm Hg in cream)		
Frequent user (n = 1)	21	n.a.
Non-user (n = 3)	9 (28)	2.5–52
**Family 2 (2013)** (96,000–210,000 ppm Hg in cream)		
Frequent user (n = 1)	208	n.a.
Infrequent user (n = 1)	14	n.a.
Non-user (n = 9)	13 (7)	6–31
**Family 3 (2010)** (56,000 ppm Hg in cream)		
Frequent user (n = 2)	252	132–482
Non-user (n = 3)	18 (56)	ND-107
**Combined Cases, GM (GSD)**		
Frequent user (n = 4)	129 (197)	37–482
Infrequent user (n = 1)	14	14
Non-user (n = 15)	13 (27)	ND-107

Note: cr. = creatinine; ND = not detected; limit of quantification = 0.5 µg/g cr. Non-detected values were assigned a value of 0.25 µg/g cr. for the purpose of calculating the geometric mean. n.a. = not applicable.

### 3.2. Environmental Assessment and Remediation

Emergency response scenarios for each family unit described above were similar. Emergency responders from US Environmental Protection Agency (US EPA) [15]. Emergency Response Section, California Department of Toxic Substances Control, or the local environmental health departments were onsite. Mercury vapor surveys were conducted in the home of each index case and in homes of identified contacts who also used the creams. 

Several state and national regulatory agencies have identified risk and action levels for assessing inorganic mercury in contaminated homes (Table 3). The Agency for Toxic Substances and Disease Registry (ATSDR) and the US EPA recommends site-specific indoor air action levels for the home and personal belongings [12]. Two action (exposure) levels are generally used to determine health risk and need for action or for evacuation: (1) 1 µg/m^3^—the indoor air level below which is considered safe for residential occupancy with no remediation required, and (2) 10 µg/m^3^—the level above which isolation from exposure or evacuation of residents is recommended (Table 3). Concentrations between 1 and 10 µg/m^3^ are recommended for remediation, without requiring evacuation of the home. In 2010, the 10 µg/m^3^ level was also used as the acceptable level for personal items to remain in the owner’s possession [13]. This level was changed to 3–6 µg /m^3^ in 2012 by ATSDR and US EPA [12]. 

**Table 3:** Health-Based Risk and Action Levels for Elemental Mercury in air.

Health-Based Risk Air levels—Elemental Hg	Value (µg/m^3^)
Evacuation Level (ATSDR)	10
Action Level (ATSDR)	1.0
Acute 1hr REL (CalEPA OEHHA)	0.6
CA 8hr REL (CalEPA OEHHA)	0.06
Intermediate MRL (ATSDR)	0.2
Chronic RfC (USEPA)	0.3
CA Chronic REL (CalEPA OEHHA)	0.03

Note: RfC = Reference Exposure Concentration; Intermediate MRL = Minimum Risk Level (exposure duration 15–364 days); REL = Reference Exposure Level (continuous exposure up to a lifetime).

### 3.3. Remedial Measures 

Face creams as the primary source of mercury were removed from the homes. Mercury levels in indoor air were further reduced by heating and ventilating the home, and using garden sulfur to decontaminate expensive personal items and washing machines. Garden sulfur was also used to decontaminate the hands of the index case in 2010 because measurements exceeded 6 µg/m^3^ after multiple washings with soap and water. Sulfur powder is commonly used in the cleanup of mercury spills [16]. Following remedial measures, air measurements were repeated throughout the home and in bagged items. All personal items showing residual mercury contamination above the action level were removed from the residence and disposed of properly. 

Due to the severity of the mercury poisoning and the presence of young children living in the 2013 and 2014 described households, investigators used more conservative action levels than those recommended by ATSDR for addressing elemental mercury spills [12]. When possible, an action level of 1 µg/m^3^ was used as the trigger for remediation of personal items belonging to the children and the index case. Given the dual air and dermal exposure pathways, the targeted goal was reduction of mercury concentrations in ambient air to 0.2 µg/m^3^, the ATSDR intermediate Minimal Risk Level [12]. 

During initial home visits, mercury levels in breathing zones ranged from non-detect to 8.0 µg/m^3^ bedrooms recorded the higher levels. (Table 4). The highest mercury vapor levels were found on the user’s hands (230 µg/m^3^) and the jars of creams (999 µg/m^3^). Mercury vapor was also measured on personal items such as clothing and beauty products; levels ranged from 0.3 µg/m^3^ to 200 µg/m^3^. 

**Table 4:** Range of mercury vapor (Hg) levels in three households.

Location or Items	Hg Vapor Range ^a^ in Homes (µg/m^3^)
Bedroom Breathing Zones	0.50–8.0
Bedding	1–200 ^b^
Clean Clothing	0.3–20
Dirty Clothing	17–200
Washers	6–11
Dryers	0.1–1.0
User’s Hands	6–230
Jars of Cream	12–999 ^c^
Bagged Personal Items (stuffed animals, remote controls, backpacks, make-up brushes)	1.7–127

Note: ^a^ All measurements were taken with either the Jerome J405 Mercury Vapor Analyzer, the Lumex RA-915 +, or the Lumex^®^ RA-915 Light; ^b^ Maximum limit of detection for the Lumex^®^ RA-915 Light. Actual mercury vapor levels are presumably higher; ^c^ 999 µg/m^3^ is the maximum limit of detection for the Jerome J405. Actual mercury vapor levels were presumably higher.

Re-assessments of homes between two days and one month after remedial measures revealed a significant reduction in mercury levels in breathing zones (non-detectable to 0.3 µg/m^3^). Following multiple cycles of sulfur treatment, mercury vapor levels measured in washing machines dropped from 11 µg/m^3^ to below 0.7 µg/m^3^. Mercury levels on the hands of the 2010 index case were reduced to 0.2 µg/m^3^ after repeated daily washing with sulfur over a one-month period [13]. 

The home of Family 2 (2013) underwent extensive mercury vapor monitoring and remedial efforts. Twelve home visits were conducted over a span of two months with more than 300 mercury vapor measurements taken throughout the home and from personal items. Post remedial efforts, children’s items not reduced to below 1 µg/m^3^ were disposed of due to concerns of potential re-exposure of a symptomatic child following hospitalization. Table 4 provides a summary of notable measurements prior to remedial efforts. 

## 4. Discussion and Conclusions

Our findings support previously known information but also provide new insight into how exposure to inorganic mercury may impact human health. Consistent with our findings, neuropsychiatric signs and symptoms have been previously described as the most sensitive signs of inorganic mercury intoxication in users of mercury-containing creams [17]. In their study, McRill *et al*. [17] reported headache, weakness, depression, dizziness, worry or anxiety, fatigue, and irritability to be the most common symptoms associated with mercury skin cream use. Back, joint and limb pain, and personality change were the next most commonly reported symptoms. 

The kidney is generally regarded as a sensitive target organ for mercury toxicity [18]. However, we found minimal clinical signs of kidney damage in cases that displayed prominent neuropsychiatric symptoms. Since signs of irritability, insomnia, and short-term memory impairment are non-specific diagnostic indicators of mercury exposure, these early signs and symptoms may not prompt appropriate recognition of mercury exposure and toxicity or the need for clinical treatment. 

Our findings identified hypertension as a common clinical sign in several exposed infants and children. Hypertension as a key diagnostic sign of mercury exposure or toxicity is not frequently discussed in the current literature, but our findings suggest greater emphasis should be placed on urinary mercury testing as a diagnostic tool in the face of unexplained hypertension in young age groups. In case reports from the 1930s and 1940s, when calomel preparations were common in teething powders and worm tablets, hypertension, often accompanied by tachycardia, was a common finding in children with “calomel disease”. In 38 case reports of children aged 10 months to five years, 25 (67%) children demonstrated signs of hypertension [19]. 

Our report highlights important information about the use patterns of mercury-containing creams. Younger, adolescent age groups were found in our study to use these creams for acne treatment. For example, in addition to the 17-year-old index case in 2013, we identified a 14-year-old adolescent male cream user in 2010. In 1995, a severely poisoned 15-year-old adolescent male was the index case among 330 individuals in an outbreak of mercury poisoning resulting from a commercial Mexican cream [20]. This use for the treatment of acne broadens the public health concern and understanding of the diverse motivations that drive people to use these creams, thereby increasing the potential number of people exposed to mercury. 

Understanding the exposure pathways and associated factors allowing mercury to enter the body are important factors to consider when setting protective exposure limits. The ATSDR toxicological profile for mercury describes a limited ability for inorganic mercury salts to cross the skin for systemic absorption [21,22]. This conclusion is supported by empirical evidence obtained under experimental conditions. In studies using human cadaver skin, Sartorelli [23] and colleagues similarly reported that dermal absorption of mercury, either using mercurous chloride, or in the form of mercury-contaminated soil, was poorly and slowly absorbed [23]. However, some studies report that absorption through the skin, particularly when well hydrated, is in fact a significant route of systemic exposure to inorganic mercury [24]. Palmer and colleagues found that mercurous chloride from cosmetic creams rapidly traversed human cadaver skin *in vitro* [25]. Daily applications of mercury-containing skin lightening cream to the back of the neck in mice, resulted in dose-dependent accumulation of mercury in the ovaries [5]. A 1947 study by Laug *et al.* [26] found calomel uptake by rats was significantly enhanced by propylene glycol as a carrier, in comparison to water, petrolatum, mineral oil, or corn oil. Consistent with these studies, our reported clinical cases provide evidence that uptake of mercurous chloride through skin creams is rapid and accompanied by significant systemic toxicity. 

It remains unknown if, beyond the simple hydration effect of these creams, specific components directly enhance mercury absorption. It is clear that once absorbed, mercury can remain in the skin for days or weeks and provide a source of contamination to associated clothing, bedding, and other personal belongings. The skin has been demonstrated to harbor mercury deposits when exposed to inorganic mercury forms. Silberberg and colleagues [27] found electron-dense granules in the inter- and intra-cellular space under the dermis following exposure to aqueous mercuric chloride. 

High mercury vapor concentrations in the air space above the hands of the 2010 index case measured 6.0 µg/m^3^ approximately 12 h after application of the skin cream. Repeated hand washing with soap and water failed to reduce the mercury vapor levels above her hands during the decontamination process. She was instructed to wash with garden sulfur powder to decontaminate her hands on a daily basis. In a follow-up visit conducted one month later, the mercury concentration in the air space above her hands was reduced but still present at 0.2 µg/m^3^ [13]. The precise mechanisms by which inorganic mercury becomes volatilized from the skin are not characterized in the literature.

Other factors may also influence the chemical state of mercury in these compounds and impact the likelihood for exposure and toxicity. Although the vapor pressure of inorganic salts of mercury is low, some environmental factors appear to influence the valence state, and thus volatility, of mercury originating from salt forms. For example, UV-light has been shown to reduce mercuric chloride to calomel and then elemental mercury in experimental studies [28,29]. The formation of elemental mercury from inorganic forms is referred to as the Eder’s Reaction and can take place in the presence of low pH and UV light [29]. Awitor and colleagues reported complete dissociation of calomel into mercuric chloride and elemental mercury under experimental conditions in the temperature range of 343–453 °K (158–356 °F) [30].

(1) Hg_2_Cl_2(solid)_ ⬄ Hg_(gas)_ + HgCl_2(gas)_

The dissociation relationship in the Awitor *et al*. [30] paper was not explored at lower, ambient or room temperatures, but our data would suggest that significant dissociation can occur at these temperatures. Similar studies of this nature at lower temperatures were not found in the literature. This potential mode of mercury transport and fate within the house provides one possible explanation for the environmental distribution of mercury on household surfaces. Presumably, surface contamination becomes widespread through touching of surfaces by the cream users and through contact with contaminated clothing. The subsequent volatilization results in a multi-pathway exposure scenario with numerous opportunities for dermal contact in addition to airborne mercury exposures. Since mercury vapor and surface residue have no sensory warning properties, the widespread contamination of the home goes unnoticed by occupants.

The three homes that underwent environmental sampling demonstrate that mercury salts are spread throughout the home by the cream users and can become a source for mercury contamination on surfaces, personal belongings and in ambient air. Mercury contamination in the homes varied but was particularly extensive in Family 2 (2013). The differences in the extent of contamination may be due to the concentration of the cream used, the duration of use, and environmental conditions such as ventilation and the presence and amount of sunlight. 

Guidance developed for elemental mercury spills by ATSDR and US EPA [12] is used when responding to mercury incidents. According to these guidelines, if ambient air levels of mercury are below the recommended action level (1 µg/m^3^), further investigation and cleanup is not considered necessary. This guidance does not take into consideration additional potential exposure routes, such as dermal absorption, ingestion via hand-to-mouth transfer, or through food prepared by the cream user. Additionally, environmental screening of residences involving contaminated face creams should not be limited to breathing zone samples; contamination may be present and should be suspected on items in obscure places, such as in washing machines, closets, mattresses, cabinets or drawers. Contamination of household items can significantly raise ambient air levels in the surrounding area. For example, in Family 2, initial breathing zone measurements taken in the bathroom showed mercury at 0.3 µg/m^3^; upon opening a cabinet door where a jar of contaminated cream had previously been stored, the ambient air level in front of the cabinet quickly rose to 4.0 µg/m^3^ (Table 4). If investigators had not conducted a thorough screening of the entire residence, taking mercury measurements in closets, cabinets, and on personal belongings, several reservoirs of mercury contamination in the household would not have been identified and remediated. Washing machines in particular emitted high levels of mercury vapor and served as a contamination source for clothes. After running multiple empty washing cycles with garden sulfur, mercury levels were reduced to below 1 µg/m^3^. 

We found unexpected and widespread mercury concentrations in the air above household items stemming from the use of inorganic mercury salt-containing creams. The air concentrations of mercury likely indicate a slow environmental conversion process of the inorganic form to elemental mercury. These findings call into question whether the existing ATSDR/US EPA clean-up air standards for mercury, which are based on the volatility and environmental properties of the elemental form, are sufficiently protective of a multi-pathway exposure setting. The method of distribution of mercury through the house from calomel-containing creams is fundamentally different from that of elemental mercury, resulting in measurable air levels at or just below the action level of 1 µg/m^3^. Thus, the air levels are a source of exposure but are also indicators of persistent contamination of surfaces and an ongoing risk of exposure through dermal contact.

These case reports demonstrate the extent to which a home and its inhabitants can become contaminated from the use of mercury-containing skin creams. Described inhabitants became contaminated and developed mercury toxicity through multi-pathway exposures, including dermal uptake, inhalation, and potential ingestion. These factors should be taken into consideration when setting standards for environmental health protection and during remediation efforts in these homes.
